# Antimicrobial peptides disrupting the bacterial membrane reduce *Salmonella* colonization in chickens

**DOI:** 10.1128/spectrum.01848-25

**Published:** 2025-11-03

**Authors:** Gary Closs, Dipak Kathayat, Yosra A. Helmy, Menuka Bhandari, Gireesh Rajashekara

**Affiliations:** 1 Department of Food Science & Technology, The Ohio State University 2647, Columbus, Ohio, USA; 2 Department of Animal Sciences, Center for Food Animal Health, Ohio Agricultural Research and Development Center, The Ohio State University 57704, Wooster, Ohio, USA; 3 Richard A. Gillespie College of Veterinary Medicine, Lincoln Memorial University, Harrogate, Tennessee, USA; 4 Department of Veterinary Science, Martin-Gatton College of Agriculture, Food, and Environment, University of Kentucky 4530, Lexington, Kentucky, USA; 5 Department of Pathobiology, College of Veterinary Medicine, University of Illinois Urbana-Champaign 14589, Urbana, Illinois, USA; University of Mississippi, University, Mississippi, USA

**Keywords:** public health, food safety, chickens, antimicrobial peptides, *Salmonella*

## Abstract

*Salmonella* is a significant food safety and public health concern. Poultry products have been considered the main vehicles of *Salmonella* infections in humans. *Salmonella* being present in visually healthy poultry is suggested as the preeminent human transmission risk factor, allowing bacteria to easily transmit from eggs and poultry to humans. Antimicrobial peptides (AMPs) have been suggested as promising alternatives to current antibiotics because of their low propensity for resistance development. In this study, we tested the efficacy of *Lactobacillus rhamnosus* GG (LGG)-derived small peptides (P1-NPSRQERR, P2- PDENK, and P4-MLNERVK) against *Salmonella* Typhimurium (ST) and *Salmonella* Enteritidis (SE) both *in vitro* and in chickens. Peptides inhibited ST, SE, and other public health–relevant *Salmonella* serovars *in vitro*. The antibacterial activity of peptides is likely due to their effect on *Salmonella* membranes. Importantly, P1 and P2 effectively reduced the colonization of ST by 2.2 and 1.8 logs, respectively, in chickens at 7 days post-infection. P1, P2, and P4 possessed no adverse effect on chicken cecum microbial community, but chickens not treated and challenged with *Salmonella* showed a difference in microbial richness and evenness compared to the unchallenged chickens. Furthermore, peptides retain their activity upon exposure to heat and protease treatments—characteristics necessary for the use of antimicrobial products in the poultry industry. In summary, these small peptides can provide a promising approach to develop novel strategies for controlling *Salmonella* infections in chickens.

**IMPORTANCE:**

*Salmonella* is the most frequently reported bacterial cause of foodborne illness in the United States. Poultry products (eggs and poultry meat) have been considered the main vehicles of *Salmonella* infections in humans. There is a need for developing and implementing effective antibiotic alternatives to reduce *Salmonella* in chickens, minimize human exposures, and simultaneously contribute to alleviating antibiotic resistance. AMPs have been suggested as promising alternatives to current antibiotics because of their low propensity for resistance development. Our study showed that LGG-derived peptides (P1-NPSRQERR, P2-PDENK, and P4-MLNERVK) significantly inhibit ST, SE and other *Salmonella* serovars *in vitro* and in chickens. Overall, our results demonstrate that small peptides can facilitate the development of promising approaches to control *Salmonella* infections in poultry, thus contributing to improved food safety and public health.

## INTRODUCTION

*Salmonella* is the most frequently reported bacterial cause of foodborne illness in the United States (US) (1, 2). *S. Enteritidis* and *S. Typhimurium* are consistently among the top serovars isolated in human infection, making them serovars of public health concern (3–5). The Centers for Disease Control and Prevention (CDC) estimates that about 1.35 million infections, 26,500 hospitalizations, and 420 deaths are caused by *Salmonella* annually in the USA (6). The annual cost of medical treatment for *Salmonella*-related foodborne illnesses in the USA is estimated to be $3.7 billion (7). Poultry products (eggs and poultry meat) have been considered the main vehicles of *Salmonella* infections in humans (3, 8). Poultry, particularly chickens and turkeys, are frequently colonized with *Salmonella* without showing detectable symptoms (sub-clinical carriers/healthy carriers), through both horizontal and vertical transmission at the primary production level (3, 9, 10). Asymptomatic carriage in poultry constitutes a key vector for human *Salmonella* infection, especially via eggs and meat (11). Controlling *Salmonella* in animals is difficult because it is environmentally persistent, easily adaptable, widely distributed, and increasing in antibiotic resistance (12).

Previously, antibiotics have been widely used to control *Salmonella* infections. However, in 2012, the Food and Drug Administration (FDA) recommended the limited use of antibiotics in food-producing animals and then later included the phasing out of antibiotics in the production use for prevention and growth in food animals (FDA Guidance for Industry #209, #213). Despite the need to reduce the overuse of antibiotics to control antibiotic-resistant bacteria, the reduction in antimicrobials can subsequently lead to an increase in foodborne and avian pathogens in poultry and poultry products (13). Therefore, there is a need for developing and implementing effective alternatives to reduce *Salmonella* in chickens, minimize human exposures, and simultaneously contribute to alleviating antibiotic resistance.

Antimicrobial peptides (AMPs), such as LL-37 and hLF1-11, have been suggested as promising alternatives to current antibiotics (14–16). Their ability to inhibit antibiotic-resistant bacteria has been deemed an advantage AMPs have over antibiotics (14, 17, 18). In addition to their low propensity for resistance development, their potential as novel therapeutics is supported by their relatively small size and fast and selective antibacterial action (14). The confidence that AMPs will be better at avoiding resistance is rooted in their mechanism of action (14, 15). AMPs involve multiple low-affinity targets, which complicates bacteria’s ability to defend against a singular resistance mechanism (15). Conventional antibiotics use a high-affinity target which allows bacteria to quickly defend and display resistance (15). AMPs’ antibacterial activity involves disrupting the bacterial membranes, degrading the cell wall, and/or acting on intracellular targets after translocation into the bacterium’s cytoplasm (14, 15). Membrane depolarization, creation of pores allowing cellular contents to leak, and alteration of the lipid bilayer could all be actions caused by AMPs, leading to disruption in bacterial membrane functions (14). Unlike many current antibiotics that elicit bacterial stress pathways, such as RpoS, AMPs do not, and are thus better at avoiding bacterial mutations (19). More importantly, AMPs do not pose a threat to mammalian cells due to structural differences between microbial and mammalian membranes, particularly the lipid composition (15). AMPs carry additional advantages in addition to the broad-spectrum antibacterial properties. Some AMPs display antiviral, antifungal, and antiparasitic properties (19). AMPs also possess immunomodulatory properties, which can aid in protection while simultaneously enhancing animal health and performance (15, 19). Small or short peptides are (typically <18 residues) easier to synthesize, making them a more feasible option for use as therapies compared to other peptides (20). For this study, short or small peptides will be defined as those with 10 or fewer amino acid residues.

The objective of this study was to test the efficacy of *Lactobacillus rhamnosus* GG (LGG) derived small peptides (P1-NPSRQERR, P2- PDENK, P3-VHTAPK, P4-MLNERVK, P5-YTRGLPM, and P6-GKLSNK) against *Salmonella enterica* subsp. *enterica* serovar Typhimurium LT2 (ST) in chickens, and subsequently to test their spectrum of activity against *Salmonella enterica* subsp. *enterica* serovar Enteritidis (SE) and other serovars *in vitro*. Since *Salmonella* is a pathogen of food safety concern, we additionally tested the ability of peptides to retain inhibition qualities when heated or treated with protease, according to poultry industry standards (21). Most importantly, we aimed to identify which small peptides would be best at retaining antibacterial capabilities in chickens and to evaluate the role, if any, peptides have on the cecal microbial community of chickens. We hypothesized that short, LGG-derived peptides could selectively inhibit pathogenic *Salmonella* strains in chickens while preserving beneficial microbiota, thereby offering a scalable antimicrobial alternative for poultry systems. Our study aimed to reduce the intestinal and extraintestinal colonization of *Salmonella* in chickens to reduce the foodborne illnesses in humans.

## MATERIALS AND METHODS

### Bacterial strains and growth conditions

The bacterial strains, their growth conditions, and sources are detailed in Table S1. *Salmonella enterica* subsp. *enterica* serovar Typhimurium LT2 (ST) (Dr. John Gunn, Nationwide Children’s Hospital, Columbus, OH) was the primary strain used for determining the antibacterial properties of the antimicrobial peptides. Nalidixic acid–resistant *Salmonella enterica* subsp. *enterica* serovar Enteritidis (SE) (from the laboratory collection) was used as a secondary strain *in vitro* to confirm the antibacterial activity. Additional food safety–relevant serovars were used to confirm the spectrum of activity of the peptides.

### Peptide synthesis

LGG-derived peptides (P1-NPSRQERR, P2- PDENK, P3-VHTAPK, P4-MLNERVK, P5-YTRGLPM, P6-GKLSNK) were used because of LGG’s ability to exhibit bactericidal activity against gram-negative bacteria, including *Salmonella* (22–25). All six peptides were synthesized using GenScript (NJ, USA) with >95% purity, dissolved in 100% dimethyl sulfoxide (DMSO), and stored at −80°C until experiments were conducted.

### Primary screening for anti-*Salmonella* activity of peptides

The inhibitory effect of the peptides was assessed as described previously (23). ST was grown overnight and adjusted to 5 × 10^5^ CFU/ml, and 100 µL of this measured concentration was placed into the wells of a 96-well plate. Peptides P1–P6 were added to the well at 12 mM concentration, and the plate was incubated in a TECAN Sunrise^TM^ absorbance microplate reader at 37°C, with kinetic absorbance measurements taken every 30 min for 12 h. The growth inhibitory activity was calculated with the formula (OD_600_ DMSO-treated well − OD_600_ peptide-treated well)/OD_600_ DMSO-treated well × 100%. Wells treated with DMSO and containing *Salmonella*, as well as sterile media wells without *Salmonella*, were used as controls. At least two independent experiments were conducted.

### Determination of minimum inhibitory concentration using dose-response analysis

To determine the minimum inhibitory concentration (MIC) of P1, P2, P3, and P4 (selected based on results of primary screening), the methodology of the assay above was used, but the peptides were added at 6 mM, 12 mM, 15 mM, and 18 mM concentrations. MIC was determined as the peptide concentration at which no increase in growth (no increase in OD) was observed, indicating complete inhibition of ST growth. Additionally, to test the efficacy of peptides against another serovar, the assay was conducted using SE, with peptides added at 15 mM and 18 mM concentrations. Following incubation (37°C, 12 h), cultures were plated on the LB agar plate to confirm the bactericidal effect. At least two independent experiments were conducted.

### Peptide spectrum of activity against varied *Salmonella* serovars

To further test the spectrum of the peptides’ anti-*Salmonella* capabilities, eight additional *Salmonella* serovars commonly implicated in foodborne illness (*S*. Anatum, *S*. Albany, *S*. Brenderup, *S*. Javiana, *S*. Heidelberg, *S*. Muenchen, *S*. Newport, *S*. Saintpaul) were investigated (Table S1). P1, P2, and P4 (selected based on dose-response assay results) were added to the assay at their MICs to determine their effect on different serovars. Inhibition was determined using the criteria described above.

### Heat and protease tolerance of peptides

P1, P2, and P4 were heated for 6 min at 86°C (21) or treated with protease proteinase K (200 µg/ml) at 50°C for 30 min (26). Afterward, the treated and/or heated peptides at their respective MICs were evaluated in the inhibition assay with *Salmonella*. The protocol from above using the TECAN microplate reader was followed with the same controls.

### Peptides’ effect against biofilm-embedded *Salmonella*

To analyze the ability of the peptides (P1, P2, and P4) to retain inhibitory characteristics against biofilm-protected *Salmonella*, Innovotech’s MBEC Assay (Innovotech Inc., AB, Canada) was conducted as previously detailed (23, 27). Initially, 150 µL of 5 × 10^7^ CFU/mL adjusted ST or SE suspension in LB media was aliquoted into the wells of MBEC inoculator plates containing polystyrene pegs and incubated for 36 h on a rocker platform at 37°C to allow the biofilm formation. After biofilm formation, the pegs were washed to remove loosely adherent planktonic bacteria and then transferred to a new 96-well plate containing peptides at MICs, with relevant controls, in 200 µL of 25% LB media. The diluted LB media was used because it allows for slow bacterial growth, promoting biofilm formation due to limited nutrients (28). The plate was incubated for 18 h at 37°C with rotation at 150 rpm. Sterile media and DMSO were used as controls. Following incubation, the MICs of peptides in the challenge plate were recorded. The pegs were transferred to a new 96-well plate containing PBS and sonicated for 30 min to disrupt the biofilm. The sonicated suspensions were then tenfold serially diluted and plated on LB agar plates to enumerate the biofilm-protected bacteria and determine the minimum biofilm eradication concentration (MBEC) of peptides. At least two independent experiments were conducted.

### Peptide activity against commensal bacteria

Peptides P1, P2, and P4 were used to assess if they would inhibit commensal microbes. The commensal bacteria used along with their culture requirements are described in Table S1. Peptides at their MICs were added to 100 µL of a known concentration of commensal bacteria and incubated under their required conditions. OD_600_ of cultures was measured to assess the effect of peptides on bacterial growth.

### Efficacy of peptides in chickens

To assess the efficacy of peptides against ST in chickens, one-day-old specific pathogen-free (SPF) layer chickens from a *Salmonella*-free flock at The Ohio State University were obtained and provided with feed and water *ad libitum*. Chickens (*n* = 10/group) were treated with P1, P2, or P4 at a dose of 50 mg/kg body weight, administered twice daily. Peptides were dissolved in water to reach a final concentration of 50 mg/kg and were orally administered (gavage) from day 1 to day 7. Early intestinal colonization is important for poultry health, and thus, we begin the peptide treatment at day 1 (29). Birds were challenged with 10^4^ CFU of nalidixic acid–resistant ST on day 3. At day 10 (7 days post-infection [dpi]), chickens were euthanized, and the cecum, liver, and spleen were collected to determine the peptide’s ability to reduce ST infection. The cecum was suspended in PBS, homogenized, tenfold serially diluted, and plated on XLT4 agar plates supplemented with 50  µg/mL nalidixic acid and incubated for 24 h at 37 °C. The liver and spleen were individually suspended in PBS, and the undiluted homogenized suspension was plated directly and incubated for 24 h at 37°C. One milliliter of the undiluted homogenized liver and spleen was enriched in 9 mL of tetrathionate broth (TTB) and incubated for 24 h at 37°C. After incubation, they were plated as described above to determine the number of birds positive for ST in each tissue. The body weights of chickens were measured on the day of necropsy (day 10). An untreated positive control group (PC: infected but untreated) and a negative control group (NC: non-infected and non-treated) were included in the study. A Mann-Whitney test was used to determine statistical significance (*P* < 0.05) of treatment on ST load in cecum and on body weight.

### Cecal microbiome analysis

To evaluate the impact of P1, P2, and P4 (50 mg/kg) treatment on the cecal microbiome of chickens, a 16S rRNA-based metagenomic study (*n* = 10/group) was conducted, as previously described (23, 30, 31). DNA was extracted from approximately 0.2 g of cecal content using PureLinkTM Microbiome DNA Purification Kit (Thermofisher Scientific). RNA was removed by treating samples with RNase A (2 µL of 100 mg/mL solution per sample; Qiagen). NanoDrop 2000c Spectrophotometer (ThermoFisher Scientific) was used to check the quality and measure the quantity of the DNA. The extracted DNA was used in 16S rRNA V4-V5 sequencing done at MCIC (https://mcic.osu.edu/genomics/illumina-sequencing). Amplicon libraries were prepared using IFU KAPA HiFi HotStart ReadyMix PCR Kit (Roche). PCR products were cleaned using Agencourt AMPure XP beads (BECKMAN COULTER Life Sciences). Nextera XT DNA Library Preparation Kit (Illumina) was used to generate the Illumina library, and sequencing was performed on the Illumina MiSeq platform, generating paired-end 300 bp reads. QIIME 2 (Quantitative Insights Into Microbial Ecology) bioinformatics platform (https://qiime2.org/) was used to conduct metagenomic analysis (32). Quality control of the raw reads was performed using FastQC 0.11.8 (Babraham Bioinformatics). Trimmomatic-0.33 was used to trim the adapter and other Illumina-specific sequences (http://www.usadellab.org/cms/?page=trimmomatic). Trimmed sequences (fastq.gz) were then imported into QIIME 2 as a manifest file format (PairedEndManifestPhred33V2). DADA2 was then used for the feature table construction and additional filtering of sequences (33). Taxonomic analysis was performed using Naive Bayes classifiers trained on the Silva 132 99% OTUs (silva-132-99-nb-classifier.qza) database. Phylogenetic diversity was analyzed using the align-to-tree-mafft-fasttree pipeline. Additionally, Shannon’s diversity index (alpha diversity) and Bray-Curtis distance (beta diversity) were analyzed using core-metrics-phylogenetic pipeline (https://docs.qiime2.org/2019.7/tutorials/moving-pictures/). Alpha and beta diversity statistical significance were analyzed using Kruskal-Wallis and PERMANOVA tests (*P* < 0.05), respectively. The statistical difference (*P* < 0.05) in the taxonomic composition between the groups was determined using the Mann-Whitney test.

### Peptide resistance studies

To assess *Salmonella*’s ability to develop resistance to P1 and P2, lethal and sub-lethal resistance assays were performed as described previously (23, 27, 34). For lethal resistance assay, approximately 10^8^ CFU of ST was plated on LB agar mixed with 5× MIC of P1 and P2 peptides and incubated for 5 days at 37°C. Sterile plates and a DMSO-treated *Salmonella* plates were used as controls. For sub-lethal resistance assay peptides (P1 and P2) were added at sub-inhibitory (0.75× MIC) concentrations to 100 µL of ST suspension (~5 × 10^5^ CFU/mL) in LB medium in 1.5 mL microcentrifuge tubes. The tubes were incubated at 37°C with shaking at 125 rpm for 24 h. After incubation, 20 µL of grown ST culture was mixed with 80 µL of fresh LB medium, and peptides were added again at 0.75× MICs. This procedure was repeated 13 times. Following the 14th passage, the MIC of peptides was determined against ST cultures grown from the 14th passage as described above. The tubes containing sterile LB medium and DMSO-treated ST suspension were used as controls. The lethal and sub-lethal resistance assays were done in duplicates.

### Structural activity relationship (SAR) study

Structure activity relationship analysis was used to determine the amino acids crucial for anti-*Salmonella* activity of P1 and P2. Alanine scanning libraries of P1 and P2 were synthesized from GenScript (Table S2; https://www.genscript.com/alanine_scanning.html) and tested for inhibitory activity (35) as described above. Relative importance [(percent growth in analogue − percent growth in original peptide) / (percent growth in DMSO-treated control −percent growth in original peptide) × 100] was used to identify the amino acids crucial for the peptide activity. At least two independent experiments were conducted.

To analyze the perceived efficacy of arginine and lysine substitutes reported in previous studies (36, 37), specific amino acid residues in P1 and P2 were replaced with lysine or arginine and tested if the substitutions enhanced anti-*Salmonella* (ST and SE) activity. The MICs of the arginine or lysine substituted peptides (NP**R**RQERR, NPSR**R**ERR, NP**R**R**R**ERR, **K**DENK, PDE**K**K, **K**DE**K**K) were determined as described above. At least two independent experiments were conducted.

### Mode of action of peptides analyzed through confocal microscopy

To determine the mode of action of P1 and P2, bacterial cytological profiling was conducted using confocal microscopy as described in (23, 27). One hundred microliters of ST (5 × 10^8^ CFU/mL) were treated with 5× MIC of P1 and P2 and then immediately incubated at 37°C with 180 rpm shaking for 3 h. Incubated bacteria cultures were then centrifuged and resuspended in 100 µL sterile water. Following suspension, 1 µg/mL of FM4-64 (Invitrogen Molecular Probes) and 5 µM Syto-9 (Invitrogen Molecular Probes) stains were added and incubated for 45 min at 4°C with shaking at 150 rpm. After incubation, the samples were centrifuged, and the bacteria were resuspended in 10 µL sterile water. For microscopic analysis, 3 µL of bacterial suspension was added to glass slides with 1.2% agar and 20% LB media. Untreated ST bacterial culture was used as a control. A Leica TCS SP6 confocal scanning microscope (excitation/emission [nm]: FM4-64 [515/640], SYTO-9 [485/498]) was used at The Ohio State University’s Molecular and Cellular Imaging Center (MCIC; https://mcic.osu.edu/microscopy).

### Transmission electron microscopy

To further visually analyze the inhibitory effects of P1 and P2 on ST, transmission electron microscopy (TEM) was performed as described previously (23, 27, 38). Briefly, 500 µL of 1 × 10^9^ CFU/mL ST was treated with 10× MIC of peptides and incubated as described above (37°C with 180 rpm shaking for 3 h). Afterward, cells were fixed overnight with 3% glutaraldehyde and 2% paraformaldehyde at room temperature. Cells were then washed and postfixed with 1% osmium tetroxide (OsO_4_). The washed cells were then dehydrated, with graded ethanol, and embedded with Embed 812 kit (Electron Microscopy Sciences, PA). Ultrathin (70 nm) sections were prepared on formvar-carbon–coated grids and stained with uranyl acetate and lead citrate. Untreated ST was used as a control. TEM was conducted at MCIC using the Hitachi H-7500 microscope.

### Docking studies

HPEPDOCK (39) and PEP-SiteFinder (40) tools were used to predict the binding affinities/propensities of peptides to OmpC (PDB 3UU2) and OmpF (PDB 3NSG) proteins of *Salmonella*. We selected OmpC and OmpF proteins because we showed previously that these peptides are likely to function by affecting the outer membrane lipid asymmetry (MlaA-OmpC/F) system in avian pathogenic *Escherichia coli* (23).

## RESULTS

### Peptides display anti-*Salmonella* activity

**Fig 1:**
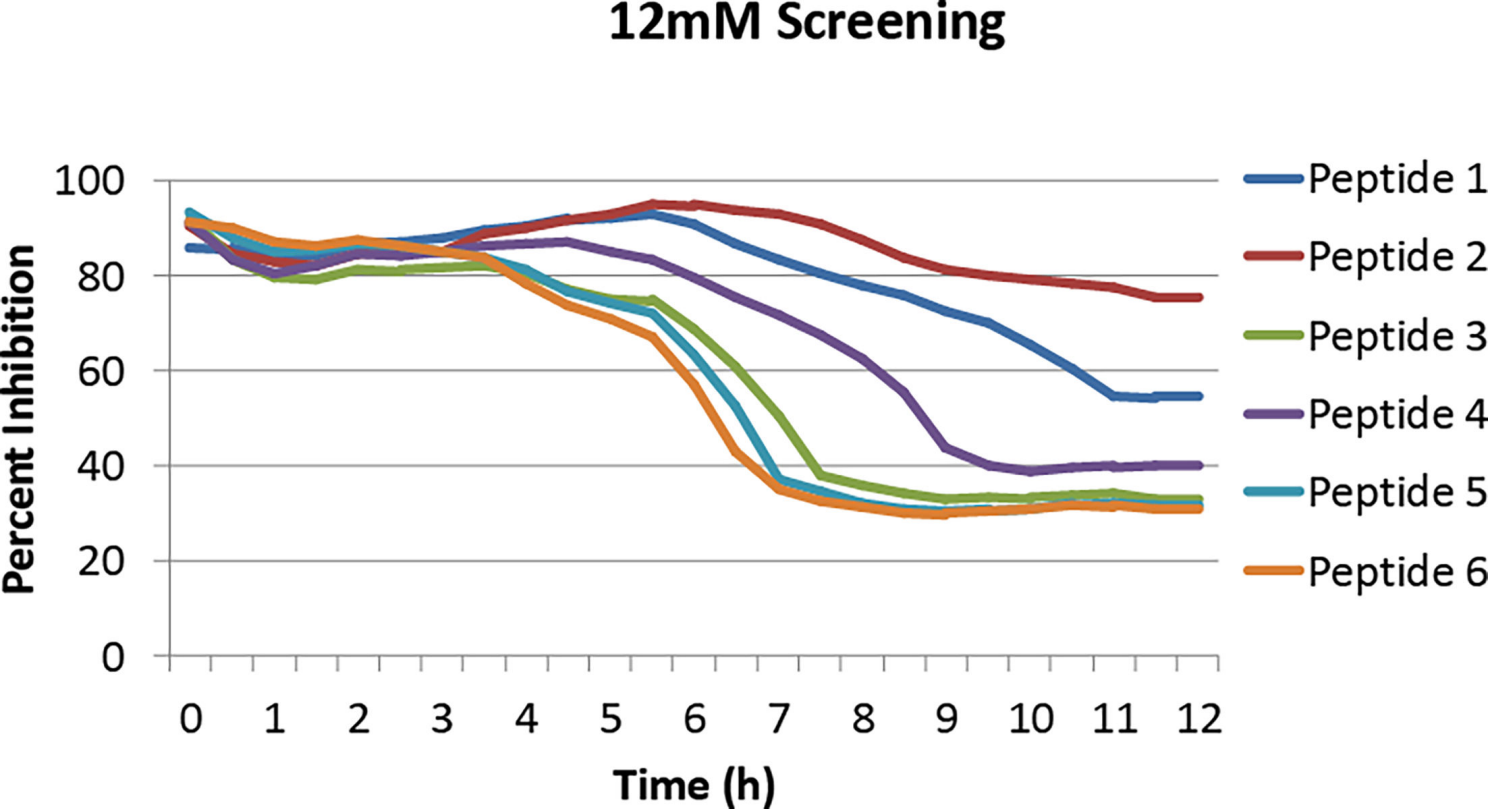
Percent inhibition of *S.* Typhimurium (ST) growth by peptides at 12 mM. Peptides were added to the wells of a 96-well plate containing ST suspension and incubated at 37°C in a TECAN Sunrise absorbance microplate reader with kinetic absorbance measurement set at every 30 min for 12 h. The inhibition (%) was calculated using the formula (Percent inhibition = (OD_600_ DMSO-treated well − OD_600_ peptide-treated well)/OD_600_ DMSO-treated well × 100%). Two independent experiments were conducted, and the average was plotted.

Peptides (P1, P2, P3, P4, P5, P6) were added to ST at 12 mM concentrations and incubated for 12 h at 37°C. The initial screening showed four peptides (P1, P2, P3, and P4) effective in inhibiting ST growth (Fig. 1). At 12 mM, P2 inhibited ST by 75%, P1 by 55%, P4 by 40%, and P3 by 33% (Fig. 1).

**Fig 2:**
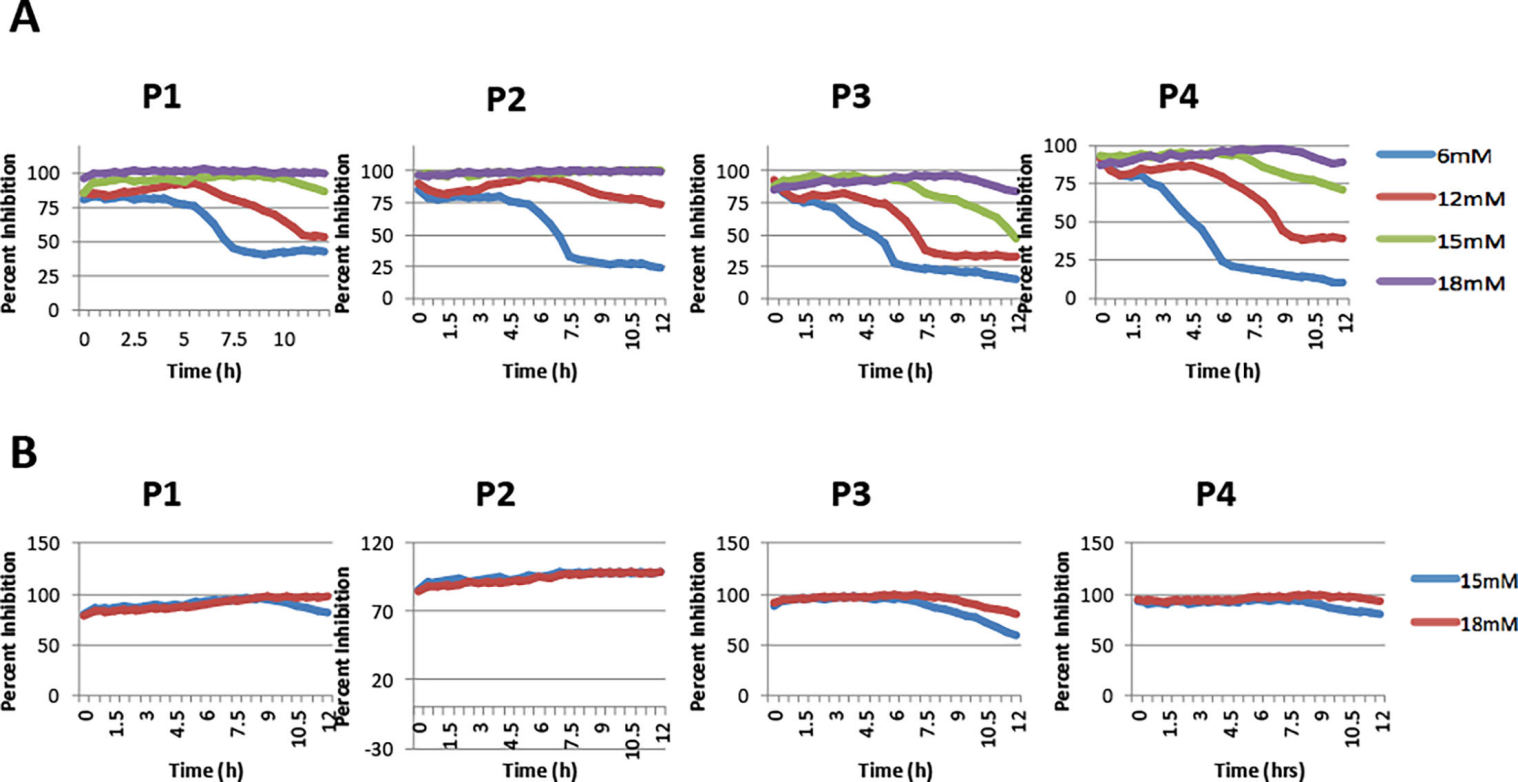
Dose response analysis showing percent inhibition of *S.* Typhimurium (ST) and *S.* Enteritidis (SE). (**A**) ST growth when treated with peptides at 6 mM, 12 mM, 15 mM, and 18 mM. (**B**) SE growth when treated with peptides at 15 mM and 18 mM. Plates were incubated in TECAN Sunrise absorbance microplate reader for 12 h at 37°C. Percent inhibition = (OD_600_ DMSO-treated well − OD_600_ peptide-treated well)/OD_600_ DMSO-treated well × 100%. Two independent experiments were conducted.

To further determine the minimum inhibitory concentration of peptides, a dose-response analysis was conducted using peptides P1, P2, P3, and P4 at 6 mM, 12 mM, 15 mM, and 18 mM concentrations, respectively. P1 and P2 completely inhibited the growth of ST at 18 mM and 15 mM, respectively (Fig. 2A). Neither P3 nor P4 completely inhibited ST; however, P4 resulted in ~90% inhibition at 18 mM (Fig. 2A). Furthermore, they were also tested against SE at 15 mM and 18 mM. P1 and P2 showed 100% inhibition at 18 mM and 15 mM, respectively (Fig. 2B). P4 resulted in 95% inhibition of SE growth at 18 mM. P3, similar to ST results, did not inhibit more than 85% of SE growth and thus was not chosen for further analyses (Fig. 2B). Based on these results, the MICs of P1, P2, and P4 were determined to be 18 mM, 15 mM, and 18 mM respectively (Fig. 2).

### Peptides retain inhibition against other serovars

P1, P2, and P4 inhibition against public health–relevant serovars ST and SE led us to test their spectrum of activity at their previously determined MICs against other non-typhoidal *Salmonella* serovars that are commonly implicated in foodborne illnesses. Table 1 shows that all serotypes tested (*S.* Anatum, *S.* Albany, *S.* Brenderup, *S.* Javiana, *S.* Heidelberg, *S.* Muenchen, *S.* Newport, *S.* Saintpaul) were inhibited completely by P1, P2, and P4 at their respective MICs. This supports the idea that the peptides have broad spectral inhibitory activity against multiple *Salmonella* serovars from varied sources.

**TABLE 1:** Efficacy of peptides against multiple *Salmonella* serovars

*Salmonella* spp.	P1	P2	P4
*Salmonella* Anatum	18 mM	15 mM	18 mM
*Salmonella* Albany	18 mM	15 mM	18 mM
*Salmonella* Brenderup	18 mM	15 mM	18 mM
*Salmonella* Javiana	18 mM	15 mM	18 mM
*Salmonella* Heidelberg	18 mM	15 mM	18 mM
*Salmonella* Muenchen	18 mM	15 mM	18 mM
*Salmonella* Newport	18 mM	15 mM	18 mM
*Salmonella* Saintpaul	18 mM	15 mM	18 mM

### Peptides are heat- and protease-resistant and inhibit biofilm-protected *Salmonella*

P1, P2, and P4 were exposed to 86°C for 6 min or were treated with protease to determine if either had an effect on the anti-*Salmonella* ability of the peptides. Neither heat nor the protease treatment had any effect on the MIC of the peptides (Table 2).

**TABLE 2:** Effect of heat and protease treatment on anti-*Salmonella* (ST and SE) activities of peptides

Peptide	MIC (mM) against ST			MIC (mM) against SE		
	No treatment	Heat treatment	Protease treatment	No treatment	Heat treatment	Protease treatment
P1	18	18	18	18	18	18
P2	15	15	15	15	15	15
P4	18	18	18	18	18	18

 Additionally, P1, P2, and P4 retained inhibitory characteristics against biofilm-protected *Salmonella*. Specifically, all viable biofilm-protected *Salmonella* (both ST and SE) were eradicated in the wells treated with peptides at their MICs (Table 3). In the MBEC assay, the untreated wells yielded 6.46 ± 0.09 log CFU/mL biofilm-protected ST. Similarly, untreated wells yielded 6.77 ± 0.02 log CFU of biofilm-protected SE.

**TABLE 3:** Inhibitory effect of peptides against biofilm-protected *Salmonella* (mean ± SD)

Peptide	Biofilm-embedded ST Log CFU/mL	Biofilm-embedded SE Log CFU/mL
P1	0.00 ± 0.00^*a*^	0.00 ± 0.00^*a*^
P2	0.00 ± 0.00^*a*^	0.00 ± 0.00^*a*^
P4	0.00 ± 0.00^*a*^	0.00 ± 0.00^*a*^
Untreated	6.46 ± 0.09	6.77 ± 0.02

^*a*^ *P* < 0.001 in Student’s *t*-test.

### Peptides have no effect on gram-positive commensal bacteria

P1, P2, and P4 were tested at their respective MICs against both gram-positive and gram-negative commensal bacteria to further understand their inhibitory characteristics. No growth inhibition was observed against any gram-positive commensal bacteria (*Enterococcus faecalis*, *Streptococcus bovis*, LGG, *Lactobacillus acidophilus*, *Lactobacillus brevis*, *Bifidobacterium lactis* Bb12, *Bifidobacterium longum*, and *Bifidobacterium adolescentis*) when treated with any of the peptides. Similarly, the peptides showed no inhibition on the growth of gram-negative bacteria *Bacteroides thetaiotaomicron*. However, *E. coli* Nissle 1917 and *E. coli* G58-1 were inhibited, suggesting that P1, P2, and P4 retained inhibition characteristics against the closely related gram-negative bacteria.

### Peptides reduce ST colonization in the chicken cecum

**Fig 3:**
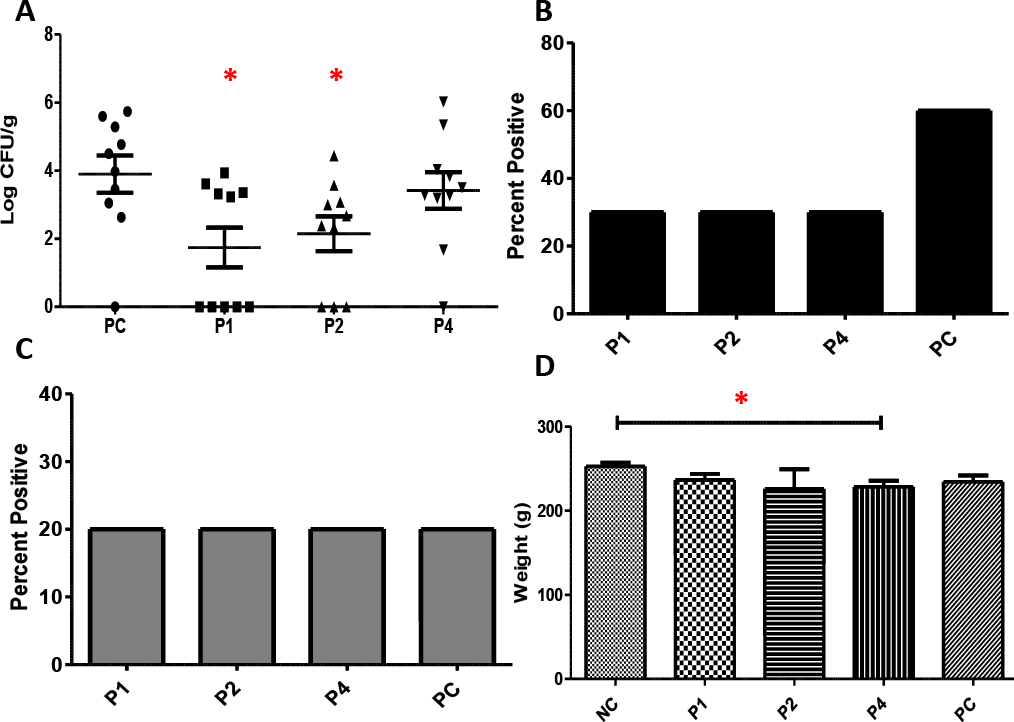
Efficacy of peptides against *S.* Typhimurium (ST) in SPF layers (*n* = 10/group) treated with peptide at 50 mg/kg per body weight twice daily for 7 days. (**A**) Effect of treatment on the reduction of ST in cecum. (**B**) Effect of peptide treatment on percent of birds positive for ST in liver enriched with TTB. (**C**) Effect of peptide treatment on percent of birds positive for ST in spleen enriched with TTB. (**D**) Effect of peptide treatment on bird weight at day 10. PC = ST challenged but untreated positive control. *P < 0.05 in Mann-Whitney test

One-day-old SPF layers were treated with P1, P2, or P4 with 50 mg/kg twice daily for 7 days to test their efficacy against *Salmonella* infection. Since both ST and SE showed consistent and similar results *in vitro*, only ST was used in the chicken experiment as a proof of concept. At day 10 (7 days post-ST infection), P1-NPSRQERR and P2-PDENK significantly reduced the colonization of ST in the cecum (Fig. 3A) by 2.2 logs and 1.8 logs, respectively (*P <* 0.05*;* Mann-Whitney test), reductions considered epidemiologically relevant in terms of lowering pathogen shedding and transmission. Additionally, all peptides reduced the percentage of birds positive for ST in the liver by 30% (Fig. 3B). There was not consistent ST colonization in the spleen, so no difference was observed between peptide-treated groups and untreated (PC) group (Fig. 3C). Furthermore, treatment with P1 or P2 did not affect the body weights of chickens (Fig. 3D).

### Peptides do not affect cecal microbial richness and diversity.

**Fig 4:**
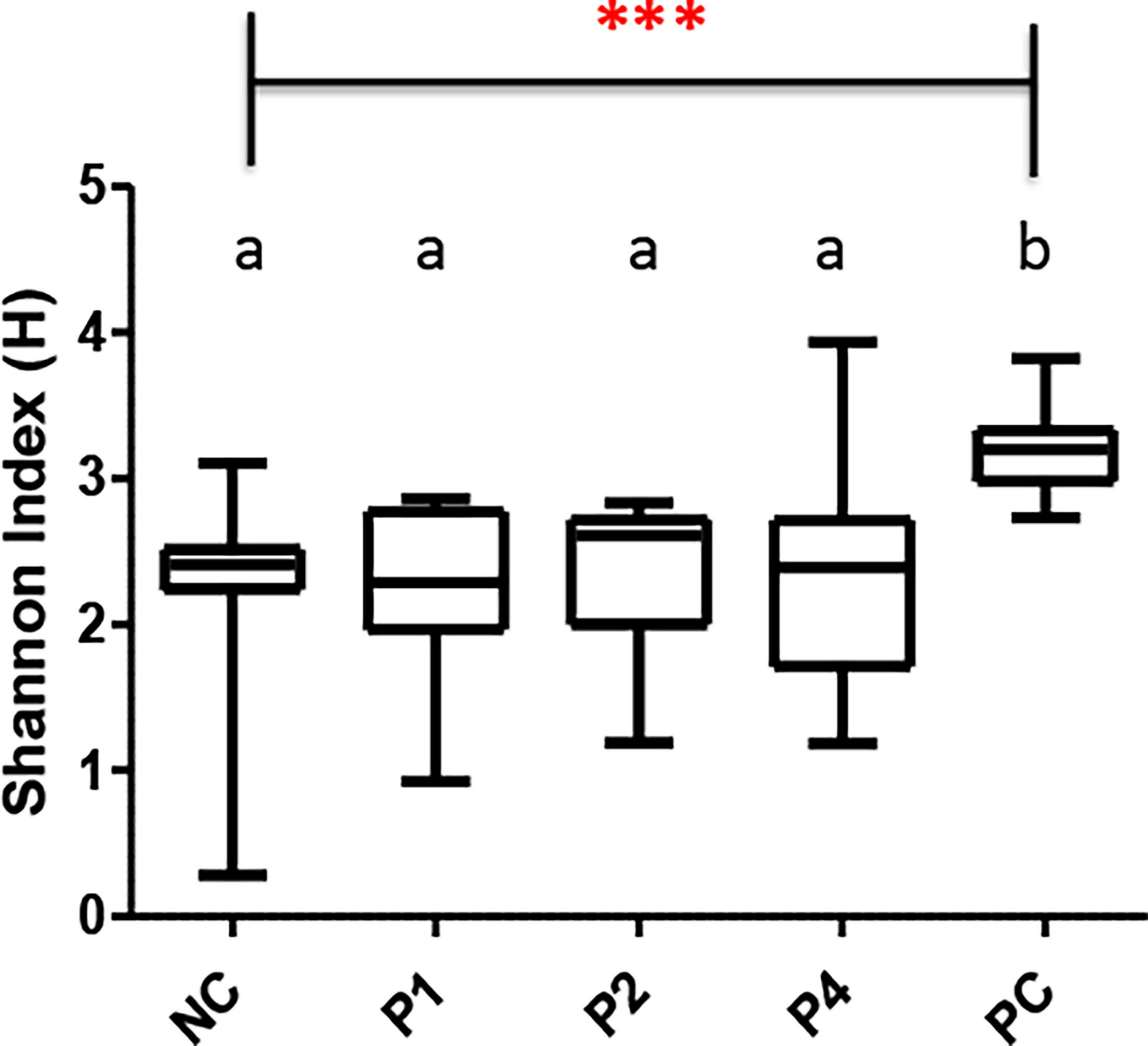
Shannon’s diversity index corresponding for alpha diversity measuring microbial richness and evenness in the cecum of chickens treated with peptides at 50 mg/kg per body weight twice daily (*n* = 10/group). NC = untreated, not challenged negative control. PC = challenged but untreated positive control. ***P < 0.001 in Kruskal-Wallis test. Different letters denote significant difference between groups.

**Fig 5:**
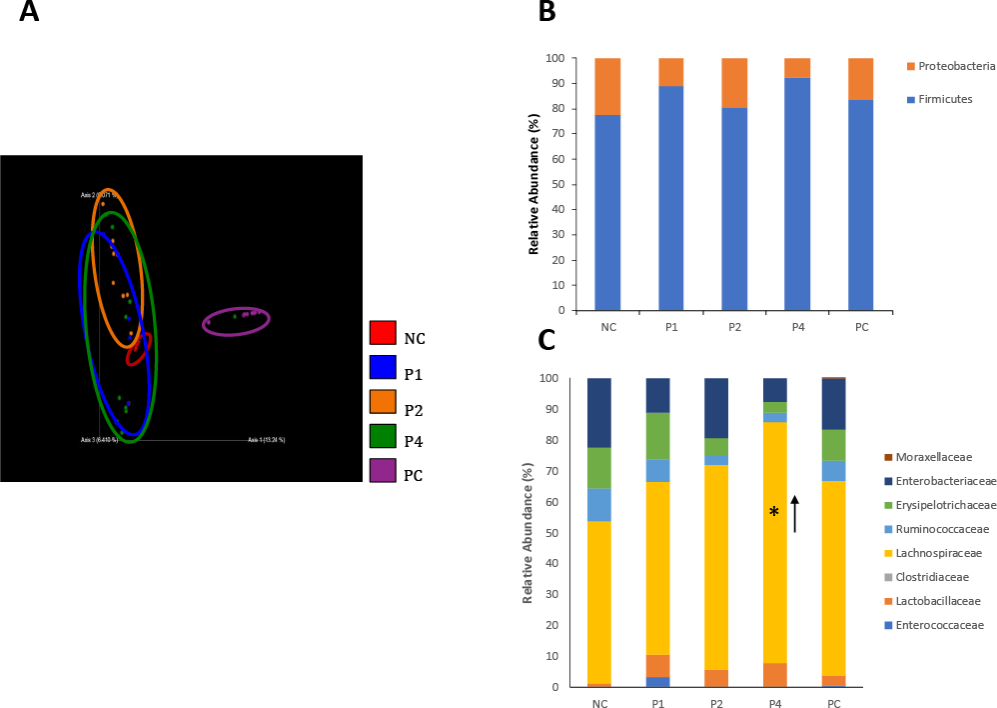
Analysis of cecal microbial community (**A**) Bray-Curtis distance plot analyzing beta diversity comparing cecum microbial communities by group. Microbial relative abundance in chicken cecum at the phylum level (**B**) and family level (**C**). NC = untreated, not challenged negative control. PC = challenged but untreated positive control. *P < 0.05 in Mann-Whitney test, (*n* = 10/group).

Analysis of alpha diversity was done using the Shannon index to determine the difference in microbial richness and diversity. Peptide-treated birds showed no significant difference compared to the non-treated, non-infected control (NC) (Fig. 4). However, NC and all peptide-treated groups showed significant differences in microbial richness and diversity compared to the *Salmonella*-infected positive control (PC; infected but untreated) group (*P < 0.001*) (Fig. 4). Similarly, all peptide-treated groups and NC group showed significant differences when compared to PC group in beta diversity (*P < 0.001*) (Fig. 5A).

At the phylum level, cecal microbiota was primarily composed of Proteobacteria and Firmicutes. Among the groups, there were no significant differences in the relative abundance at the phylum level (Fig. 5B). Similarly, P1, P2, and PC showed significant differences in relative abundance at the family level. However, P4 did significantly increase the relative abundance of *Lachnospiraceae* from 53% to 78% compared to the negative control (Fig. 5C). At the genus level, P4 (11.71%) and PC (4.91%) significantly increased the *Lachnospiraceae* (uncultured) compared to NC (0.37%) (Table S3). P1 (4.80%) and P4 (3.16%) reduced the percentage of pathogenic (25) *Escherichia-Shigella* when compared to PC (11.61%).

### Glutamate (E) and aspartate (D) are important in *Salmonella* inhibition

**Fig 6:**
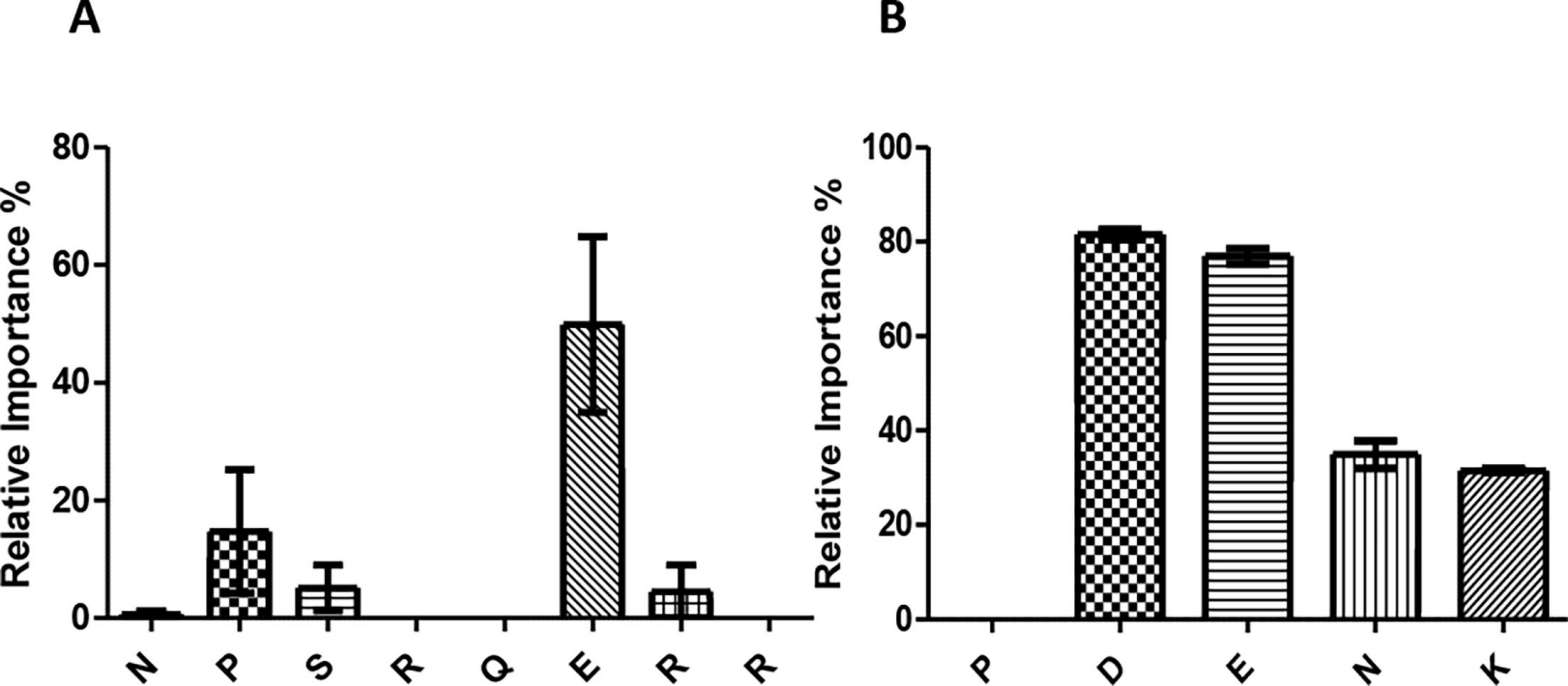
Relative importance of amino acid residues in (**A**) P1 and (**B**) P2 using alanine scanning. Relative importance was calculated using the formula: (percent growth in analogue − percent growth in original peptide) / (percent growth in DMSO-treated control − percent growth in original peptide) × 100.

Following their efficacy in chickens, P1 (NPSRQERR) and P2 (PDENK) were investigated further to determine the important amino acid residues necessary for anti-*Salmonella* activity. The complete list of alanine-substituted amino acids with its correlating sequence and peptide is listed in Table S2. In P1, glutamate (E) was the most essential amino acid with a mean relative importance of 50% (Fig. 6A). Proline (P; 15%) was the next amino acid in the ranking order of importance; however, like the remaining residues, it displayed a relative importance of less than 20%. This suggests that in P1, the ranking of amino acids is E > P > R = S (Fig. 6A). For P2, there was more of a distinct ranking of amino acids. Aspartate (D) was the most essential amino acid with a relative importance of 82%, immediately following glutamate (E) with 77% (Fig. 6B). Glutamate (E) shows high importance in both peptides, suggesting it may be essential in *Salmonella* inhibition. Asparagine (N) was the third most important residue with 35% relative importance, followed by lysine (K) with 32% (Fig. 6B). This information suggests that the relevant importance of residues in P2 is D > E > N > K. Proline (P) was the only amino acid residue in P2 to display zero relative importance, suggesting it is non-essential for anti-*Salmonella* activity (Fig. 6B). The alanine scanning analyses were only done with ST, and both ST and SE consistently showed the same growth and inhibitory characteristics, suggesting that the same amino acids would be relevant for SE.

### Lysine substitution improved anti-*Salmonella* activity of P2

The arginine (R) or lysine (K) substitutions were made in each peptide to enhance the anti-*Salmonella* activity (36, 37). Arginine showed inconsistent results against *Salmonella* when substituted for other amino acid residues (Table 4). Particularly, when arginine replaced the non-essential amino acid serine, it improved the efficacy of P1 against ST, lowering MIC from 18 mM to 15 mM (Table 4). This is in contrast to the observed change in MIC when arginine replaced glutamine (from 18 mM to >18 mM). No arginine substitution improved the MIC of the P1 against SE (Table 4). However, lysine improved the efficacy of P2 against ST and SE when it was used to replace the non-essential amino acid proline (Table 4). In both instances, the MIC improved from 15 mM to 12 mM (Table 4). Replacing asparagine with lysine did not alter a change in MIC (Table 4).

**TABLE 4:** MICs of arginine (R) and lysine (K) substituted peptide analogs

Peptide	*Salmonella* serovar^*a*^	MIC
NPSRQERR	ST	18 mM
NP**R**RQERR	ST	15 mM
NPSR**R**ERR	ST	>18 mM
NP**R**R**R**ERR	ST	15 mM
NPSRQERR	SE	18 mM
NP**R**RQERR	SE	18 mM
NPSR**R**ERR	SE	>18 mM
NP**R**R**R**ERR	SE	>18 mM
PDENK	ST	15 mM
**K**DENK	ST	12 mM
PDE**K**K	ST	15 mM
**K**DE**K**K	ST	15 mM
PDENK	SE	15 mM
**K**DENK	SE	12 mM
PDE**K**K	SE	12 mM
**K**DE**K**K	SE	15 mM

^*a*^ ST, *Salmonella *Typhimurium*;* SE, *Salmonella *Enteritidis.

### Microscopy images show peptides likely to disrupt the *Salmonella* outer membrane

**Fig 7:**
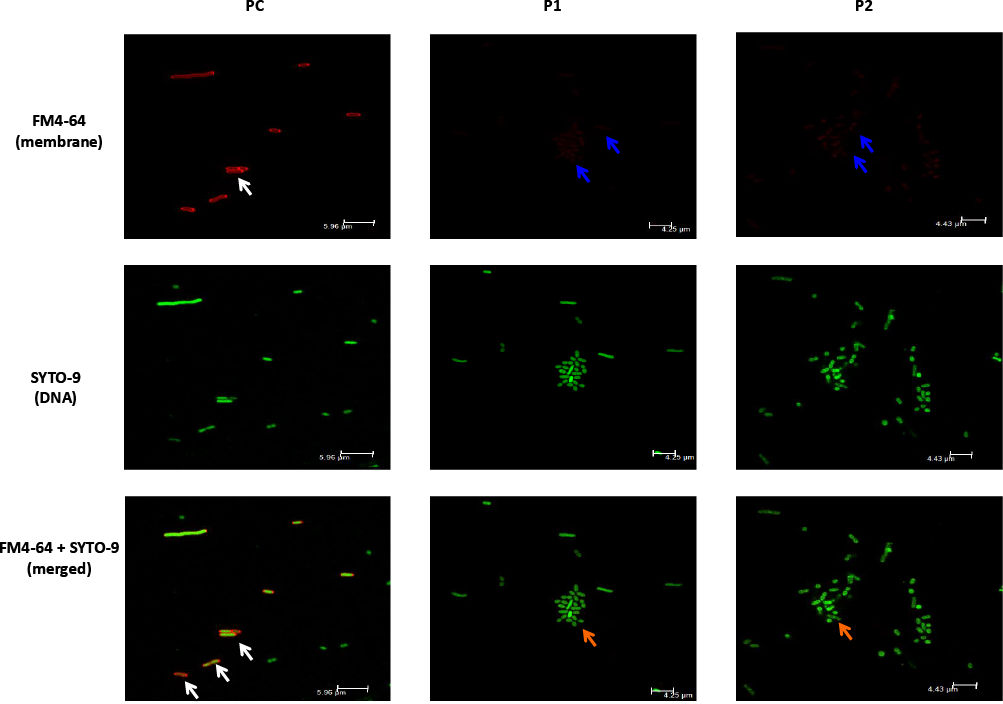
Confocal microscopy images of untreated and peptide-treated *S.* Typhimurium (ST). ST was treated with 5× peptide MIC, incubated for 3 h, and stained for 45 min with red FM4-64 membrane and green nucleic acid SYTO-9 stains. *Salmonella* membrane was clearly visible in the PC sample (white arrows), whereas no or minimally (blue arrows) visible membrane was observed in peptide-treated samples. Superimposed images (FM4-64 plus SYTO-9) showed nuclear material of *Salmonella* enclosed by membrane in PC (white arrows), whereas no membrane was visible (orange arrows) covering the nuclear material in peptide-treated cultures.

To better understand the mode of action of peptides P1 and P2, bacterial cytological profiling was done using confocal microscopy (41, 42). Only P1 and P2 were included for microscopic studies due to their promising efficacy both *in vitro* and *in vivo*. The red FM4-64 membrane stain revealed that the untreated ST (PC) had a defined outer membrane enclosing bacterial contents (Fig. 7). There was little to no red stain present in the P1- and P2-treated bacteria. Additionally, when FM6−64 + SYTO-9 (green colored DNA stain) stains were merged, the untreated ST showed DNA content surrounded by a defined red outer membrane, which is absent in the peptide-treated bacteria (Fig. 7).

**Fig 8:**
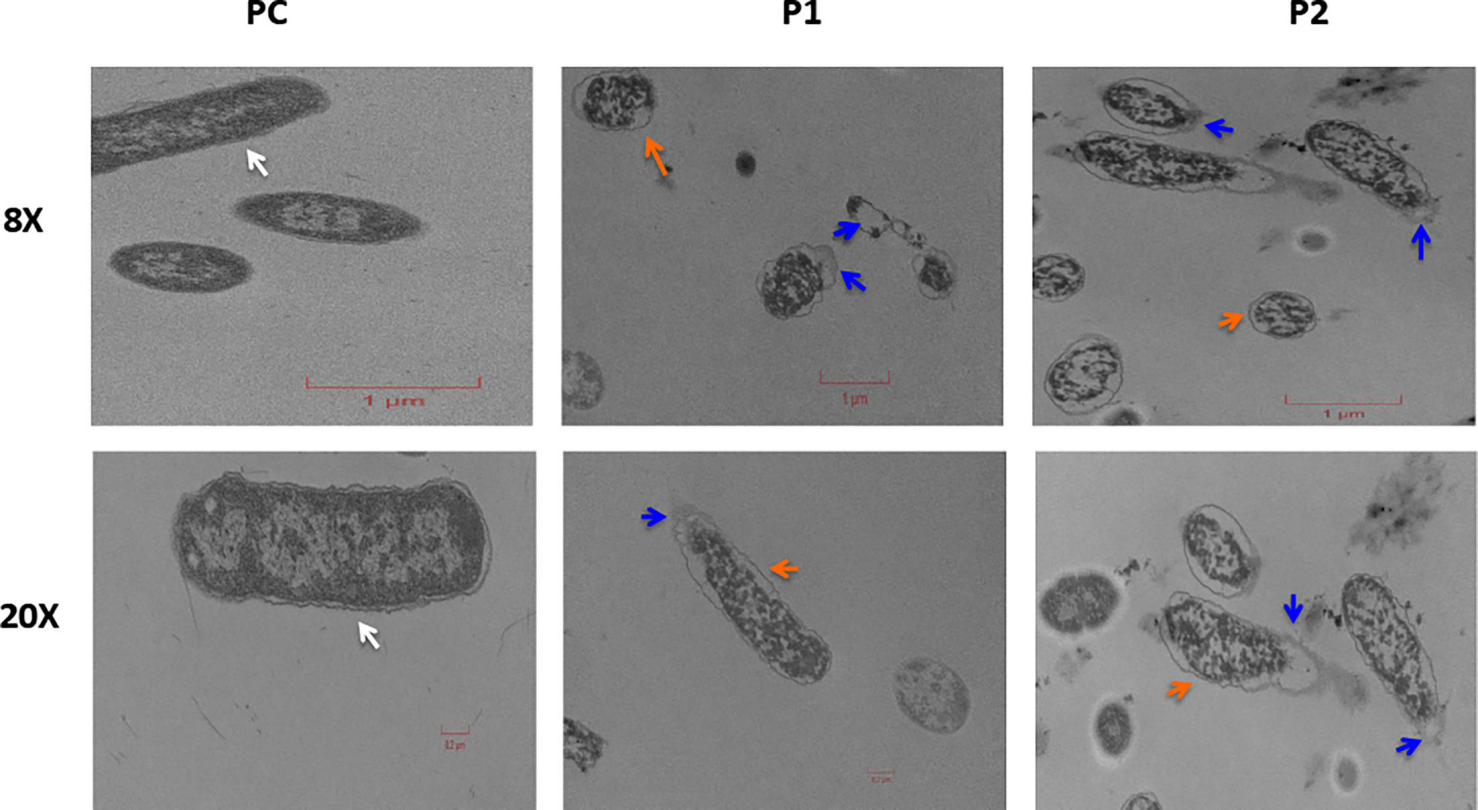
Transmission electron microscopy images of untreated and peptide-treated *S.* Typhimurium (ST). ST was treated with 10× peptide MIC and incubated for 3 h. Images displayed at 8× and 20× magnification. A clearly demarcated membrane encircling the dense cytoplasmic contents was observed in untreated *S.* Typhimurium (white arrows), whereas the membrane was either sloughed/shed (blue arrows) or flaccid (orange arrows) in ST treated with peptides. Scale bars: 1 µm (8×), 0.2 µm (20×).

The TEM images further confirmed the results from confocal images. The P1- and P2-treated samples show a disruption of the outer membrane characterized by a sloughed or flaccid appearance of the membrane (Fig. 8). In a distinct contrast, untreated ST (PC) shows an intact bacterial outer membrane (Fig. 8).

Our binding affinities prediction showed that P1 and P2 bind with higher affinities to OmpF (−209.43 and −155.62 kcal/mol, respectively) compared to OmpC (−207.37 and −141.33 kcal/mol, respectively). P1 binds with highest propensities (43–45) at Y97, Y101, and S105 residues of the B chain of OmpF, whereas P2 binds with the highest propensities (27) at Y36, E58, Y97, D108, M109, A110, and Y301 residues of the A chain of OmpF. P1 binds with the highest propensities (43) at E66 residue of the B chain and at L20, T22, D26, G28, Y35, Q59, Q61, W72, E109, F110, A117, and G342 residues of the C chain of OmpC. P2 binds with the highest propensities (17) at V100 and V106 residues of the A chain, R124 residue of B chain, and G189, G190, A191, I192, V218, Y219, T220, G221, G222, Y236, and Y316 residues of the C chain of OmpC.

## DISCUSSION

This study demonstrates for the first time that LGG-derived short peptides (<10 residues) exhibit robust anti-*Salmonella* activity both *in vitro* and *in vivo*, including biofilm eradication and microbiota preservation. *Salmonella* remains a significant food safety and public health issue (1, 3). This can be partly attributed to the increasing antibiotic resistance observed (3, 46, 47). There has not been much progress in introducing new and clinically relevant classes of antibiotics that are effective against gram-negative bacteria without having issues with rapid resistance development (48, 49). The outer membrane of gram-negative bacteria has added to the difficulty in discovering new and effective antimicrobials. The tight packing of lipopolysaccharides and the negative charge help gram-negative bacteria evade most hydrophobic molecules (49). This also suggests that targeting the outer membrane of gram-negative bacteria can aid in inhibition; however, there are not many antibiotics that can successfully do so, and those that are approved have very narrow therapeutic index and are associated with other deleterious effects (50). Previously, studies have shown that probiotics and their derived peptides possess anti-*Salmonella* capabilities (26, 43–45, 51, 52). Moreover, our previous studies confirmed that the small peptides derived from LGG are major contributors to this inhibitory effect (23, 24). In this study, we set out to test the efficacy of LGG-derived small peptides (P1-NPSRQERR, P2-PDENK, P4-MLNERVK) as potential antibacterial candidates against *Salmonella* in chickens and if they can offer antagonistic abilities against multiple serovars *in vitro*.

Our study shows that small peptides (<10 amino acid residues), a phenomenon not thoroughly studied in the literature, have an antagonistic effect against *Salmonella*. There are, however, promising studies evaluating larger peptides and their roles against gram-negative bacteria (16, 53). Particularly, peptides Alyserin (identified in secretions from frog skin) and A3APO (from synthetic peptide library) were tested against both *E. coli* and *Salmonella* and showed inhibition, but did not negatively affect *L. lactis* (16). This is similar to what we observed: peptides in our study were inhibitory against gram-negative bacteria (including commensal *E. coli*) but had no effect on gram-positive beneficial bacteria. This antagonist effect was heat- and protease-resistant, necessary characteristics for use in commercial feed settings (21).

Antimicrobial peptides have also been shown to have antagonistic effects against pathogenic bacteria in murine models (17, 50, 54). In one single treatment, the antimicrobial peptide SAAP-148 peptide was effective as a topical ointment against methicillin-resistant *Staphylococcus aureus* (MRSA) and multidrug-resistant *A. baumannii* in mice and in *ex vivo* human skin model (17). Similarly, AA139 reduced bacteria load by multiple logs in murine models challenged with *P. aeruginosa* and multidrug-resistant *E. coli* (50). Although there are not extensive studies analyzing the inhibitory effects of antimicrobial peptides against gram-negative bacteria in chickens, some studies have examined the role of peptides on broiler growth and performance (53, 55, 56). Collectively, the preliminary analyses in chicken models and bacterial inhibition observed in murine models suggest that antimicrobial peptides are considerable options for implementation in chickens. Our study confirms this: P1 and P2 significantly reduced the amount of ST in chickens by 2.2 and 1.8 logs, respectively (Fig. 3A). Additionally, P1, P2, and P4 reduced the colonization of ST in the liver compared to the untreated positive control (Fig. 3B). These peptides were only tested at 50 mg/kg as a proof of concept; future testing at higher doses may show improved efficacy. However, the synthesis cost of peptides and the delivery feasibility are the current limitations.

Consistent with the efficacy of the SAAP-148 against biofilm bacteria (17), P1, P2, and P4 completely eliminated biofilm-protected SE and ST (Table 3). These studies collectively suggest that antimicrobial peptides are valid candidates to treat antibiotic-resistant gram-negative pathogens. Given the importance of antimicrobial resistance, we tested the ability of *Salmonella* to develop resistance against lethal and sub-lethal treatment of peptides and observed no resistance. We hypothesized that the likely mode of action of the peptides disrupting the bacterial outer membrane is the reason for the lack of resistance (Fig. 7 and 8). Currently, outer membrane–targeting peptides, such as Zosurabalpin and OMN6, are in the clinical antibacterial pipeline (57). Furthermore, the preclinical membrane–targeting antibiotic SCH79797 is reported to be less susceptible to bacterial resistance (58). These findings support previous studies suggesting that resistance is less likely to develop when the mode of action targets the outer membrane (16, 59). Studies investigating the synergistic effect of our outer membrane targeting peptides with antibiotics may help increase efficacy and reduce resistance to those antibiotics. Further studies need to be conducted to accurately determine the potential target(s) of peptides, elucidate their mechanism of action, and robustly evaluate their resistance acquisition potential.

Peptides (P1, P2, and P4) had no effect on the richness and evenness of chicken cecal microbiota. However, the untreated, *Salmonella*-infected chickens did affect the alpha diversity of the microbiota. It is significant and worth noting that the peptides in our study caused no deleterious effect on microbiota differing from antibiotics that have been associated with immune dysfunction and the development of autoimmune disease (29). Antibiotic treatments have also been linked to increasing susceptibility to intestinal pathogens (29). Future chicken trials should be conducted with broilers over an extended period of time and in conditions that mimic field settings (oral delivery of peptides in water or feed) to assess the peptides’ impact on cecal microbiota at an early age, as well as their impact on performance parameters, such as body weight gain and feed conversion ratio.

In conclusion, LGG-derived peptides (P1-NPSRQERR, P2-PDENK, and P4-MLNERVK) significantly inhibit ST, SE, and other *Salmonella* serovars *in vitro*. Furthermore, the antimicrobial properties of the peptides were unaffected by heat and protease treatment, suggesting it is possible to incorporate them in commercial feed. These findings support the possible integration of LGG-derived short peptides into poultry feeding strategies as a novel, resistance-limiting intervention aligned with global antibiotic stewardship goals.

## SUPPLEMENTAL MATERIAL

Supplemental material spectrum.01848-25-s0001.docx Tables S1 to S3.

ASM does not own the copyrights to Supplemental Material that may be linked to, or accessed through, an article. The authors have granted ASM a non-exclusive, world-wide license to publish the Supplemental Material files. Please contact the corresponding author directly for reuse.

## References

[1] Scallan E, Hoekstra RM, Angulo FJ, Tauxe RV, Widdowson MA, Roy SL, Jones JL, Griffin PM. 2011. Foodborne illness acquired in the United States--major pathogens. Emerg Infect Dis 17:7–15. doi:10.3201/eid1701.p11101 21192848 PMC3375761

[2] CDC. 2018. Estimates of foodborne illness in the United States.

[3] Antunes P, Mourão J, Campos J, Peixe L. 2016. Salmonellosis: the role of poultry meat. Clin Microbiol Infect 22:110–121. doi:10.1016/j.cmi.2015.12.004 26708671

[4] CDC. 2016. National Enteric Disease Surveillance: Salmonella annual report, National Center for Emerging and Zoonotic Infectious Diseases (U.S.). Division of Foodborne, Waterborne, and Environmental Diseases.

[5] Foley SL, Nayak R, Hanning IB, Johnson TJ, Han J, Ricke SC. 2011. Population dynamics of Salmonella enterica serotypes in commercial egg and poultry production. Appl Environ Microbiol 77:4273–4279. doi:10.1128/AEM.00598-11 21571882 PMC3127710

[6] CDC. 2023. Salmonella. Centers for Disease Control and Prevention, National Center for Emerging and Zoonotic Infectious Diseases (NCEZID), Division of Foodborne, Waterborne, and Environmental Diseases (DFWED).

[7] BMaMB SH. 2015. Economic burden of major foodborne illnesses acquired in the United States. U.S. Department of Agriculture Economic Research Service.

[8] Batz MB, Hoffmann S, Morris JG Jr. 2012. Ranking the disease burden of 14 pathogens in food sources in the United States using attribution data from outbreak investigations and expert elicitation. J Food Prot 75:1278–1291. doi:10.4315/0362-028X.JFP-11-418 22980012

[9] Barrow PA. 2000. The paratyphoid Salmonellae. Rev Sci Tech 19:351–375. doi:10.20506/rst.19.2.1225 10935268

[10] Brito JRF, Xu Y, Hinton M, Pearson GR. 1995. Pathological findings in the intestinaltract and liver of chicks after exposure to Salmonella serotypes Typhimurium or Kedougou. British Veterinary Journal 151:311–323. doi:10.1016/S0007-1935(95)80181-2 7640959

[11] Akil L, Ahmad HA. 2019. Quantitative risk assessment model of human salmonellosis resulting from consumption of broiler chicken. Diseases 7:19. doi:10.3390/diseases7010019 30736421 PMC6473936

[12] Chen HM, Wang Y, Su LH, Chiu CH. 2013. Nontyphoid Salmonella infection: microbiology, clinical features, and antimicrobial therapy. Pediatr Neonatol 54:147–152. doi:10.1016/j.pedneo.2013.01.010 23597525

[13] Hao H, Cheng G, Iqbal Z, Ai X, Hussain HI, Huang L, Dai M, Wang Y, Liu Z, Yuan Z. 2014. Benefits and risks of antimicrobial use in food-producing animals. Front Microbiol 5:288. doi:10.3389/fmicb.2014.00288 24971079 PMC4054498

[14] Baltzer SA, Brown MH. 2011. Antimicrobial peptides: promising alternatives to conventional antibiotics. J Mol Microbiol Biotechnol 20:228–235. doi:10.1159/000331009 21894027

[15] Mahlapuu M, Håkansson J, Ringstad L, Björn C. 2016. Antimicrobial peptides: an emerging category of therapeutic agents. Front Cell Infect Microbiol 6:194. doi:10.3389/fcimb.2016.00194 28083516 PMC5186781

[16] Volzing K, Borrero J, Sadowsky MJ, Kaznessis YN. 2013. Antimicrobial peptides targeting gram-negative pathogens, produced and delivered by lactic acid bacteria. ACS Synth Biol 2:643–650. doi:10.1021/sb4000367 23808914 PMC4222081

[17] de Breij A, Riool M, Cordfunke RA, Malanovic N, de Boer L, Koning RI, Ravensbergen E, Franken M, van der Heijde T, Boekema BK, Kwakman PHS, Kamp N, et al.. 2018. The antimicrobial peptide SAAP-148 combats drug-resistant bacteria and biofilms. Sci Transl Med 10:eaan4044. doi:10.1126/scitranslmed.aan4044 29321257

[18] Lee JK, Luchian T, Park Y. 2018. New antimicrobial peptide kills drug-resistant pathogens without detectable resistance. Oncotarget 9:15616–15634. doi:10.18632/oncotarget.24582 29643997 PMC5884652

[19] Lewies A, Du Plessis LH, Wentzel JF. 2019. Antimicrobial peptides: the achilles’ heel of antibiotic resistance? Probiotics Antimicrob Proteins 11:370–381. doi:10.1007/s12602-018-9465-0 30229514

[20] Hamley IW. 2017. Small bioactive peptides for biomaterials design and therapeutics. Chem Rev 117:14015–14041. doi:10.1021/acs.chemrev.7b00522 29227635

[21] Russell C. 2019. Feed sanitation: heat treatment as a method for the decontamination of poultry feeds.

[22] Lu R, Fasano S, Madayiputhiya N, Morin NP, Nataro J, Fasano A. 2009. Isolation, identification, and characterization of small bioactive peptides from Lactobacillus GG conditional media that exert both anti-gram-negative and gram-positive bactericidal activity. J Pediatr Gastroenterol Nutr 49:23–30. doi:10.1097/MPG.0b013e3181924d1e 19465870

[23] Kathayat D, Closs G, Helmy YA, Lokesh D, Ranjit S, Rajashekara G. 2021. Peptides affecting outer membrane lipid asymmetry (MlaA-OmpC/F) system reduce avian pathogenic Escherichia coli (APEC) colonization in chickens. Appl Environ Microbiol. doi:10.1128/aem.00567-21:Aem0056721 PMC8357279 34132592

[24] Kathayat D, Closs G, Helmy YA, Deblais L, Srivastava V, Rajashekara G. 2022. In vitro and in vivo evaluation of Lacticaseibacillus rhamnosus GG and Bifidobacterium lactis Bb12 against avian pathogenic Escherichia coli and identification of novel probiotic-derived bioactive peptides. Probiotics Antimicrob Proteins 14:1012–1028. doi:10.1007/s12602-021-09840-1 34458959

[25] Closs G Jr, Bhandari M, Helmy YA, Kathayat D, Lokesh D, Jung K, Suazo ID, Srivastava V, Deblais L, Rajashekara G. 2025. The probiotic Lacticaseibacillus rhamnosus GG supplementation reduces Salmonella load and modulates growth, intestinal morphology, gut microbiota, and immune responses in chickens. Infect Immun 93:e0042024. doi:10.1128/iai.00420-24 40172512 PMC12070740

[26] Zhang Y, Zhang L, Du M, Yi H, Guo C, Tuo Y, Han X, Li J, Zhang L, Yang L. 2011. Antimicrobial activity against Shigella sonnei and probiotic properties of wild lactobacilli from fermented food. Microbiol Res 167:27–31. doi:10.1016/j.micres.2011.02.006 21466951

[27] Kathayat D, Helmy YA, Deblais L, Rajashekara G. 2018. Novel small molecules affecting cell membrane as potential therapeutics for avian pathogenic Escherichia coli. Sci Rep 8:15329. doi:10.1038/s41598-018-33587-5 30333507 PMC6193035

[28] Haney EF, Trimble MJ, Cheng JT, Vallé Q, Hancock REW. 2018. Critical assessment of methods to quantify biofilm growth and evaluate antibiofilm activity of host defence peptides. Biomolecules 8:29. doi:10.3390/biom8020029 29883434 PMC6022921

[29] Diaz Carrasco JM, Casanova NA, Fernández Miyakawa ME. 2019. Microbiota, gut health and chicken productivity: what is the connection? Microorganisms 7:374. doi:10.3390/microorganisms7100374 31547108 PMC6843312

[30] Helmy YA, Kathayat D, Ghanem M, Jung K, Closs G, Deblais L, Srivastava V, El-Gazzar M, Rajashekara G. 2020. Identification and characterization of novel small molecule inhibitors to control Mycoplasma gallisepticum infection in chickens. Vet Microbiol 247:108799. doi:10.1016/j.vetmic.2020.108799 32768201

[31] Kathayat D, Helmy YA, Deblais L, Srivastava V, Closs G, Khupse R, Rajashekara G. 2021. Novel small molecule growth inhibitor affecting bacterial outer membrane reduces extraintestinal pathogenic Escherichia coli (ExPEC) infection in avian model. Microbiol Spectr 9:e0000621. doi:10.1128/Spectrum.00006-21 34468186 PMC8557866

[32] Bolyen E, Rideout JR, Dillon MR, Bokulich NA, Abnet CC, Al-Ghalith GA, Alexander H, Alm EJ, Arumugam M, Asnicar F, et al.. 2019. Reproducible, interactive, scalable and extensible microbiome data science using QIIME 2. Nat Biotechnol 37:852–857. doi:10.1038/s41587-019-0209-9 31341288 PMC7015180

[33] Callahan BJ, McMurdie PJ, Rosen MJ, Han AW, Johnson AJA, Holmes SP. 2016. DADA2: high-resolution sample inference from Illumina amplicon data. Nat Methods 13:581–583. doi:10.1038/nmeth.3869 27214047 PMC4927377

[34] Deblais L, Helmy YA, Kathayat D, Huang HC, Miller SA, Rajashekara G. 2018. Novel imidazole and methoxybenzylamine growth inhibitors affecting Salmonella cell envelope integrity and its persistence in chickens. Sci Rep 8:13381. doi:10.1038/s41598-018-31249-0 30190570 PMC6127322

[35] Migoń D, Jaśkiewicz M, Neubauer D, Bauer M, Sikorska E, Kamysz E, Kamysz W. 2019. Alanine scanning studies of the antimicrobial peptide aurein 1.2. Probiotics Antimicrob Proteins 11:1042–1054. doi:10.1007/s12602-018-9501-0 30569430 PMC6695355

[36] Cutrona KJ, Kaufman BA, Figueroa DM, Elmore DE. 2015. Role of arginine and lysine in the antimicrobial mechanism of histone-derived antimicrobial peptides. FEBS Lett 589:3915–3920. doi:10.1016/j.febslet.2015.11.002 26555191 PMC4713009

[37] Li J, Koh JJ, Liu S, Lakshminarayanan R, Verma CS, Beuerman RW. 2017. Membrane active antimicrobial peptides: translating mechanistic insights to design. Front Neurosci 11:73. doi:10.3389/fnins.2017.00073 28261050 PMC5306396

[38] Hartmann M, Berditsch M, Hawecker J, Ardakani MF, Gerthsen D, Ulrich AS. 2010. Damage of the bacterial cell envelope by antimicrobial peptides gramicidin S and PGLa as revealed by transmission and scanning electron microscopy. Antimicrob Agents Chemother 54:3132–3142. doi:10.1128/AAC.00124-10 20530225 PMC2916356

[39] Zhou P, Jin B, Li H, Huang SY. 2018. HPEPDOCK: a web server for blind peptide–protein docking based on a hierarchical algorithm. Nucleic Acids Res 46:W443–W450. doi:10.1093/nar/gky357 29746661 PMC6030929

[40] Saladin A, Rey J, Thévenet P, Zacharias M, Moroy G, Tufféry P. 2014. PEP-siteFinder: a tool for the blind identification of peptide binding sites on protein surfaces. Nucleic Acids Res 42:W221–6. doi:10.1093/nar/gku404 24803671 PMC4086095

[41] Dean CR, Barkan DT, Bermingham A, Blais J, Casey F, Casarez A, Colvin R, Fuller J, Jones AK, Li C, Lopez S, Metzger LE 4th, et al.. 2018. Mode of action of the monobactam LYS228 and mechanisms decreasing in vitro susceptibility in Escherichia coli and Klebsiella pneumoniae. Antimicrob Agents Chemother 62:e01200-18. doi:10.1128/AAC.01200-18 30061293 PMC6153799

[42] Nonejuie P, Burkart M, Pogliano K, Pogliano J. 2013. Bacterial cytological profiling rapidly identifies the cellular pathways targeted by antibacterial molecules. Proc Natl Acad Sci USA 110:16169–16174. doi:10.1073/pnas.1311066110 24046367 PMC3791758

[43] De Keersmaecker SCJ, Verhoeven TLA, Desair J, Marchal K, Vanderleyden J, Nagy I. 2006. Strong antimicrobial activity of Lactobacillus rhamnosus GG against Salmonella Typhimurium is due to accumulation of lactic acid. FEMS Microbiol Lett 259:89–96. doi:10.1111/j.1574-6968.2006.00250.x 16684107

[44] Khan S, Chousalkar KK. 2020. Salmonella Typhimurium infection disrupts but continuous feeding of Bacillus based probiotic restores gut microbiota in infected hens. J Animal Sci Biotechnol 11:29. doi:10.1186/s40104-020-0433-7 PMC7087389 32211190

[45] Tejero-Sariñena S, Barlow J, Costabile A, Gibson GR, Rowland I. 2012. In vitro evaluation of the antimicrobial activity of a range of probiotics against pathogens: evidence for the effects of organic acids. Anaerobe 18:530–538. doi:10.1016/j.anaerobe.2012.08.004 22959627

[46] Carter A, Adams M, La Ragione RM, Woodward MJ. 2017. Colonisation of poultry by Salmonella Enteritidis S1400 is reduced by combined administration of Lactobacillus salivarius 59 and Enterococcus faecium PXN-33. Vet Microbiol 199:100–107. doi:10.1016/j.vetmic.2016.12.029 28110775

[47] Cohen ML, Tauxe RV. 1986. Drug-resistant Salmonella in the United States: an epidemiologic perspective . Science 234:964–969. doi:10.1126/science.3535069 3535069

[48] Agyare C, Zumbi CN, Osei FB, Boamah VE. 2018. Antibiotic use in poultry production and its effects on bacterial resistance. In Kumar Y (ed), Antimicrobial resistance - a global threat. IntechOpen, Rijeka.

[49] MacNair CR, Brown ED. 2020. Outer membrane disruption overcomes intrinsic, acquired, and spontaneous antibiotic resistance. mBio 11. doi:10.1128/mBio.01615-20 PMC7512548 32963002

[50] Elliott AG, Huang JX, Neve S, Zuegg J, Edwards IA, Cain AK, Boinett CJ, Barquist L, Lundberg CV, Steen J, Butler MS, Mobli M, et al.. 2020. An amphipathic peptide with antibiotic activity against multidrug-resistant gram-negative bacteria. Nat Commun 11:3184. doi:10.1038/s41467-020-16950-x 32576824 PMC7311426

[51] D. Wolfend A, .L. Vicent J, P. Higgins J, Andreatti RL, E. Higgins S, M. Hargis B, Tellez G. 2007. Effect of organic acids and probiotics on Salmonella enteritidis infection in broiler chickens. International J of Poultry Science 6:403–405. doi:10.3923/ijps.2007.403.405

[52] Yang Y, Latorre JD, Khatri B, Kwon YM, Kong BW, Teague KD, Graham LE, Wolfenden AD, Mahaffey BD, Baxter M, Hernandez-Velasco X, Merino-Guzman R, Hargis BM, Tellez G. 2018. Characterization and evaluation of lactic acid bacteria candidates for intestinal epithelial permeability and Salmonella Typhimurium colonization in neonatal turkey poults. Poult Sci 97:515–521. doi:10.3382/ps/pex311 29077972

[53] Wang S, Zeng X, Yang Q, Qiao S. 2016. Antimicrobial peptides as potential alternatives to antibiotics in food animal industry. IJMS 17:603. doi:10.3390/ijms17050603 27153059 PMC4881439

[54] Lee HR, You DG, Kim HK, Sohn JW, Kim MJ, Park JK, Lee GY, Yoo YD. 2020. Romo1-derived antimicrobial peptide is a new antimicrobial agent against multidrug-resistant bacteria in a murine model of sepsis. mBio 11:e03258-19. doi:10.1128/mBio.03258-19 32291307 PMC7157825

[55] Choi SC, Ingale SL, Kim JS, Park YK, Kwon IK, Chae BJ. 2013. An antimicrobial peptide-A3: effects on growth performance, nutrient retention, intestinal and faecal microflora and intestinal morphology of broilers. Br Poult Sci 54:738–746. doi:10.1080/00071668.2013.838746 24397510

[56] Choi SC, Ingale SL, Kim JS, Park YK, Kwon IK, Chae BJ. 2013. Effects of dietary supplementation with an antimicrobial peptide-P5 on growth performance, nutrient retention, excreta and intestinal microflora and intestinal morphology of broilers. Animal Feed Science and Technology 185:78–84. doi:10.1016/j.anifeedsci.2013.07.005

[57] Theuretzbacher U. 2025. The global resistance problem and the clinical antibacterial pipeline. Nat Rev Microbiol 23:491–508. doi:10.1038/s41579-025-01169-8 40210708

[58] Maharramov E, Czikkely MS, Szili P, Farkas Z, Grézal G, Daruka L, Kurkó E, Mészáros L, Daraba A, Kovács T, Bognár B, Juhász S, et al.. 2025. Exploring the principles behind antibiotics with limited resistance. Nat Commun 16:1842. doi:10.1038/s41467-025-56934-3 39984459 PMC11845477

[59] Lehman KM, Grabowicz M. 2019. Countering gram-negative antibiotic resistance: recent progress in disrupting the outer membrane with novel therapeutics. Antibiotics (Basel) 8:163. doi:10.3390/antibiotics8040163 31554212 PMC6963605

