# Supplementary material for: Antimicrobial peptides disrupting the bacterial membrane reduce Salmonella colonization in chickens
Source: Microbiol Spectr. 2025 Nov 3;13(12):e01848-25. doi: 10.1128/spectrum.01848-25 (PMC12671136; doi:10.1128/spectrum.01848-25)
Supplement: Supplemental material — Tables S1 to S3. [file spectrum.01848-25-s0001.docx]

**Supplementary Tables**

**Table S1**. Organism, source and growing conditions of bacteria used in this study

| Bacteria | Growing Conditions | Source |
| --- | --- | --- |
| *S.* Typhimurium (nalidixic acid resistant) | LB broth, aerobic 37°C for 18-24 hours | Dr. John Gunn,  Ohio State University |
| *S.* Enteritidis (nalidixic acid resistant) |  | Laboratory Collection |
| *S.* Anatum |  | Laboratory Collection |
| *S.* Albany |  | Laboratory Collection |
| *S.* Brenderup |  | Laboratory Collection |
| *S.* Javiana |  | Laboratory Collection |
| *S.* Heidelberg |  | Laboratory Collection |
| *S.* Muenchen |  | Laboratory Collection |
| *S.* Newport |  | Laboratory Collection |
| *S.* Saintpaul |  | Laboratory Collection |
| *Enterococcus faecalis* | MRS broth, 37°C, anaerobic for 16-18 hours | David Francis, SDSU |
| *Streptococcus bovis* | MRS broth, 37°C, anaerobic for 16-18 hours | David Francis, SDSU |
| *Lactobacillus brevis* | MRS broth, 37°C, anaerobic for 1-2 days | David Francis, SDSU |
| *Lactobacillus acidophilus* | MRS broth, 37°C, anaerobic for 24 hours | David Francis, SDSU |
| *Lactobacillus rhamnosus* GG | MRS broth, 37°C, anaerobic for 24 hours | ATCC, USA |
| *Bifidobacterium longum* | MRS broth with 0.05% cysteine, 37°C, anaerobic for 24 hours | David Francis, SDSU |
| *Bifidobacterium adolescentis* | MRS broth with 0.05% cysteine, 37°C, anaerobic for 24 hours | David Francis, SDSU |
| *Bifidobacterium lactis* Bb12 | MRS broth with 0.05% cysteine, 37°C, anaerobic for 24 hours | Christian Hansen, Ltd, Hørsholm, Denmark |
| *Escherichia coli* *Nissle* 1917 | LB broth, aerobic 37°C for 12-18 hours, 180 rpm | Dr. Ulrich Sonnenborn, Ardeypharm GmbH, Herdecke, Germany |
| *Escherichia coli* G58- 1 | LB broth, aerobic 37°C for 12-18 hours, 180 rpm | David Francis, SDSU |
| *Bacteroides thetaiotaomicron* | MRS broth, 37°C, anaerobic for 4-5 days | David Francis, SDSU |

**Table S2**. Peptide sequence with amino acids substituted in alanine scanning analysis

| Peptide | Sequence | Amino acid substituted |
| --- | --- | --- |
| P1-1 | **A**PSRQERR | Asparagine |
| P1-2 | N**A**SRQERR | Proline |
| P1-3 | NP**A**RQERR | Serine |
| P1-4 | NPS**A**QERR | Arginine |
| P1-5 | NPSR**A**ERR | Glutamine |
| P1-6 | NPSRQ**A**RR | Glutamic acid |
| P1-7 | NPSRQE**A**R | Arginine |
| P1-8 | NPSRQER**A** | Arginine |
| P2-1 | **A**DENK | Proline |
| P2-2 | P**A**ENK | Aspartate |
| P2-3 | PD**A**NK | Glutamic acid |
| P2-4 | PDE**A**K | Asparagine |
| P2-5 | PDEN**A** | Lysine |

**Table S3.** Relative abundance of cecal microbial community at the genus level by treatment group

| Genus | Relative Abundance (%) | | | | |
| --- | --- | --- | --- | --- | --- |
|  | NC | P1 | P2 | P4 | PC |
| *Enterococcus* | 0.00 | 3.32 | 0.00 | 0.00 | 0.57 |
| *Lactobacillus* | 1.02 | 7.00 | 5.57 | 7.01 | 2.92 |
| *Pediococcus* | 0.00 | 0.00 | 0.00 | 0.75 | 0.00 |
| *Clostridium sensu stricto 1* | 0.00 | 0.00 | 0.00 | 0.00 | 0.12 |
| *Blautia* | 0.00 | 0.00 | 0.00 | 1.60 | 0.00 |
| *Epulopiscium* | 0.00 | 0.00 | 0.17 | 0.00 | 0.00 |
| *Lachnospiraceae NK4A136 group* | 0.00 | 0.98 | 0.00 | 0.53 | 0.00 |
| *Sellimonas* | 2.63 | 2.68 | 1.76 | 0.57 | 2.50 |
| *[Ruminococcus] torques group* | 22.35 | 9.83 | 23.19 | 19.27 | 20.02 |
| *Lachnospiraceae (uncultured)* | 0.37 | 8.62 | 4.21 | *11.71 | *4.91 |
| *Lachnospiraceae (unidentified)* | 27.22 | 34.16 | 37.10 | 44.19 | 35.66 |
| *Anaerotruncus* | 0.00 | 0.00 | 0.00 | 0.48 | 1.83 |
| *Caproiciproducens* | 0.00 | 0.00 | 0.63 | 0.00 | 0.00 |
| *Flavonifractor* | 4.85 | 4.40 | 2.33 | 1.20 | 2.27 |
| *Oscillibacter* | 4.30 | 2.62 | 0.00 | 1.35 | 1.03 |
| *Ruminiclostridium 5* | 1.31 | 0.00 | 0.00 | 0.00 | 0.00 |
| *Ruminiclostridium 9* | 0.00† | 0.00† | 0.00† | 0.13† | 1.50 |
| *[Eubacterium] coprostanoligenes group* | 0.15 | 0.00 | 0.00 | 0.00 | 0.00 |
| *Erysipelatoclostridium* | 13.30 | 15.34 | 5.56 | 3.51 | 10.03 |
| *[Clostridium] innocuum group* | 0.00 | 0.00 | 0.00 | 0.00 | 0.08 |
| *Escherichia-Shigella* | 6.05 | 4.80† | 13.25 | 3.16† | 11.61 |
| *Enterobacteriaceae (unidentified)* | 16.46 | 6.26 | 6.22 | 4.54 | 4.87 |
| *Acinetobacter* | 0.00 | 0.00 | 0.00 | 0.00 | 0.08 |

* Significantly different from NC, *P<0.05*, two-tailed t-test

† Significantly different from PC, *P<0.05*, two-tailed t-test
